# Initial experience with robotic technology for thoracic surgery using the da Vinci Xi system in Tibet, China

**DOI:** 10.3389/fsurg.2024.1415704

**Published:** 2024-05-30

**Authors:** Ni Ping, Zhou Baoguo, Zhaxi Renqing, Wang Shaodong

**Affiliations:** ^1^ Department of Thoracic Surgery, People’s Hospital of Tibet Autonomous Region, Lhasa, China; ^2^ Department of Thoracic Surgery, Peking University People’s Hospital, Beijing, China

**Keywords:** robotic surgery, Tibetan plateau, da Vinci surgery system, minimally in vasive therapy, thoracic surgery

## Abstract

**Objective:**

Although the robotic surgical system has accumulated rich experience in the development of thoracic surgery, its application in Tibet area is relatively late. We report our experience concerning da Vinci Xi system in thoracic surgery and observe its practicability and surgical effect.

**Methods:**

We retrospectively analyzed 26 patients who underwent robotic thoracic surgery including: twelve lung resection, two esophagectomies, ten mediastinal surgeries and two rib mass resection. The data of patient characteristics, operative time, perioperative complications were collected.

**Results:**

Of the 26 patients, 22 cases were completed with da Vinci system successfully, including 7 segmentectomies, 4 lobectomies, 1 subsegmentectomy, 2 esophagectomies, 10 mediastinal surgeries (6 thymic resections, 3 posterior mediastinal tumor resection, 1 mediastinal cyst resection) and 2 rib mass resection. In which, 3 cases of lung resection begun with robotic technique were converted to thoracoscopic surgery (due to calcification of hilar lymph node), 1 case of bilobectomy was converted to thoracotomy due to thoracic adhesion. All the operations went well and no patients need blood transfusion. All patients had satisfactory postoperative recovery.

**Conclusion:**

It is safe, reliable and effective to carry out robotic thoracic surgery on the plateau. On the premise of carefully and seriously discussing the indications of surgery, we should actively carry out the application of da Vinci robotic surgery system in Tibet Plateau.

## Introduction

Da Vinci surgical robot is the most successful and widely used surgical robot in the world at present (1). In 2001, Yoshino took the lead in applying da Vinci surgical robot to perform non-invasive thymoma resection (2), which opened the precedent for the application of da Vinci surgical robot in thoracic surgery. The first robot-assisted thoracic surgery in China was completed in Shanghai Chest Hospital in 2009 (3). In recent ten years, robotic surgery has been carried out in more and more thoracic surgery in China.

With high altitude and relatively harsh natural environment, the traditional surgery in Tibet face greater risks and challenges. Through robotic surgery, doctors can operate surgical instruments more accurately, reduce injuries and bleeding during surgery, thus reducing the risk of surgery and improving the success rate of surgery (4). Meanwhile, robot surgery can assist surgeons to perform more complicated operations, improve the quality of surgery, and make more efficient use of limited medical resources. At present, the da Vinci robotic surgery system is widely used in thoracic surgery, and almost all lung, esophagus, mediastinum and diaphragm operations under traditional endoscopy can be assisted by the robotic surgery system (5). Robot surgery can also reduce doctors' work intensity and their physical burden in plateau environment, which is beneficial to doctors’ physical and mental health. However, the application and development of the robotic surgery system in Tibet is relatively backward and has not been reported.

## Methods

This study is a retrospective study, which was approved by the Ethics Committee of Xizang Autonomous Region (No.ME-TBHP-24-KJ-030), and all patients had informed consent. From July 2023 to March 2024, we performed 26 da Vinci operations, including 14 males, with an average age of 45 years. The operations included 4 lobectomies (2 cases of upper right lobe resection, 1 case of lower right lobe resection and 1 case of right middle and lower lobe resection) and 7 segmentectomies (3 cases of posterior segment resection of right upper lobe, 2 cases of superior segment resection of the right lower lobe, 1 case of lingular segment resection and 1 case of apicoposterior segment resection of the left upper lobe), 2 cases of radical resection of esophageal cancer, 10 cases of mediastinal tumor resection (6 cases of anterior mediastinum, 3 cases of posterior mediastinal tumor and 1 case of middle mediastinum) and 2 cases of rib tumor resection. The clinical data and pathological features of the patients are shown in Table 1.

**Table 1:** Clinical features of patients undergoing robotic thoracic surgery.

Age (years)/sex	Pre-operative conditions	Procedure	Pathological findings	Operative course	Discharge (days)
55/M	Unremarkable	Subsegmentectomy	Adenocarcinoma T1N0	Unremarkable	11
68/M	Diabetes	Segmentectomy	AdenocarcinomaT1N0	Unremarkable	16
34/M	Unremarkable	Segmentectomy	Inflammatory nodule	Unremarkable	7
49/F	Hypertension	Segmentectomy	AdenocarcinomaT1N0	Unremarkable	15
57/M	History of chest trauma	Segmentectomy	Foreign body	Convert to thoracoscopy	18
34/F	Hypertension	Segmentectomy	Pulmonary cyst	Unremarkable	15
53/F	History of gastrointestinal bleeding	Segmentectomy	Inflammatory nodule	Convert to thoracoscopy	21
30/M	Unremarkable	Posterior tumor resection	Neurinoma	Unremarkable	7
22/F	Pulmonary tuberculosis	Posterior tumor resection	Fibromatosis	Convert to thoracoscopy	13
52/M	Hepatitis B	Right upper lobectomy	AdenocarcinomaT1N1	Unremarkable	10
64/M	Hepatitis B	Right upper lobectomy	Pulmonary tuberculoma	Unremarkable	9
26/M	Unremarkable	Right lower lobectomy	AdenocarcinomaT1N1	Unremarkable	9
33/F	Cholecystectomy	Thymectomy	Thymic cyst	Unremarkable	8
37/F	Unremarkable	Thymectomy	Thymic hyperplasia	Unremarkable	10
25/F	Bronchial asthma	Thymectomy	Thymic hyperplasia	Unremarkable	13
24/F	Bronchiectasia	Rib mass	Chondroma	Unremarkable	19
32/F	Unremarkable	Rib mass	Fibrous dysplasia	Unremarkable	9
71/M	Chronic atrophic gastritis	Ivor lewis esophagectomy	Esophageal squamous carcinoma T3N2	Unremarkable	18
46/M	Gallstone	Posterior tumor resection	Mediastinal abscess	Unremarkable	10
48/M	History of chest trauma	Mediastinal cystectomy	Thymic cyst	Unremarkable	9
59/F	Appendectomy	Segmentectomy	AdenocarcinomaT1N0	Unremarkable	7
65/M	Spontaneous pneumothorax	Ivor lewis esophagectomy	Esophageal squamous carcinoma	Unremarkable	16
53/F	Pleural effusion	Bilobectomy	AdenocarcinomaT1N1	Convert to thoracotomy	13
52/F	Diabetes	Thymectomy	Thymic cyst	Unremarkable	8
41/M	Coxotuberculosis	Thymectomy	Thymic cyst	Unremarkable	16
38/M	Unremarkable	Thymectomy	Thymic hyperplasia	Unremarkable	7

## Operative technique

**Figure 1:**
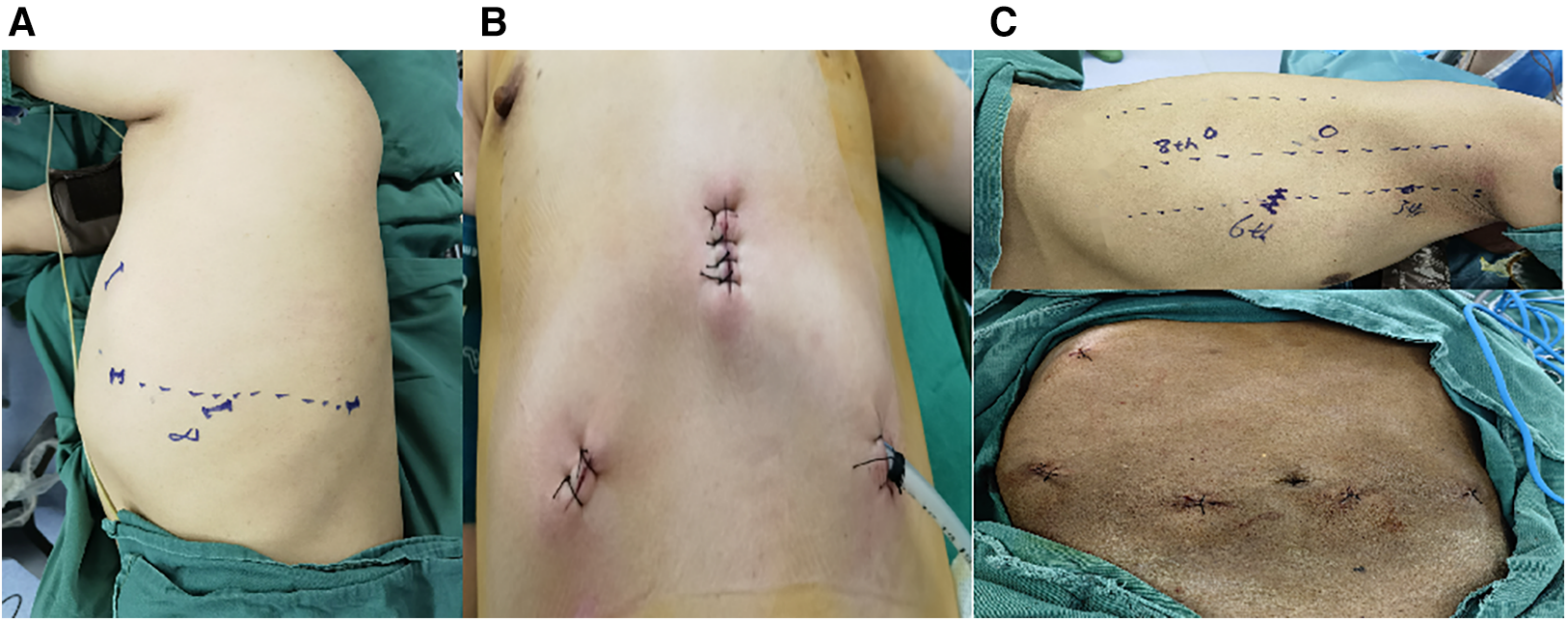
Incisions of robot-assisted thoracic surgery by means of the da Vinci Xi system. (**A**) Incisions design of pulmonary surgery and posterior tumor resection: observation port is placed in the eighth intercostal space in the midaxillary line, and the robotic arm holes are made at a horizontal interval of 6 cm beside it, and the three holes are basically a horizontal line; The auxiliary hole is about 3 cm located at the 5th intercostal axillary line. (**B**) Incisions design of subxiphoid thymectomy: a 2 cm subxiphoid incision is the observation hole, the robotic arm hole is set the lower edge of the ribs at the midclavicular line respectively. (**C**) Incisions design of esophagectomy: the 6th intercostal of the right anterior axillary line is set as the observation port, the 3th intercostal is set as the primary operation port, the 6th intercostal of the right midaxillary line is set as the secondary operation port, and the 8th intercostal of the right midaxillary line is set as the operation port for the assistant surgeon.

**Figure 2:**
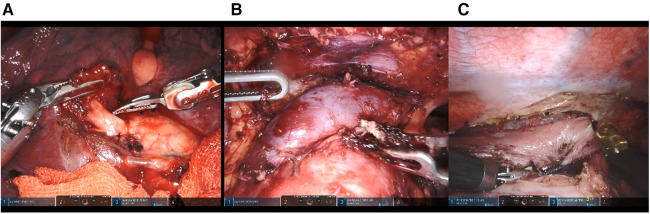
Operative pictures of robot-assisted thoracic surgery by means of the da Vinci Xi system. (**A**) Operative picture of pulmonary surgery. (**B**) Operative picture of subxiphoid thymectomy. (**C**) Operative picture of esophagectomy.

All the operations were independently performed by a chief surgeon who was familiar with thoracoscopy and thoracotomy. All the patients were performed under one-lung ventilation and general anesthesia. The position of lung resection and posterior mediastinal tumor resection was in lateral position, the robot arm system was located on the patient's back, and the placement position of four holes was shown in Figure 1A. The anterior mediastinal surgery was completed through the subxiphoid approach in supine position, the position of three holes was shown in Figure 1B. The incision distribution of esophageal surgery was shown in Figure 1C. The operative pictures of robotic sugeries were shown in Figure 2.

## Result

22 cases were successfully completed with the assistance of da Vinci robot, with an average operation time of 196 min and an average operative blood loss of 163 ml. There were no patients who need blood transfusion perioperatively. All 4 cases which converted to thoracoscopic sugery or thoracotomy were pulmonary resection, in which 1 case of lobectomy and 2 cases of segmentectomies were transferred to thoracoscopic surgery to complete the operation due to calcified hilar lymph nodes, which made pulmonary artery branches difficult to dissociate. Meanwhile one case of lung adenocarcinoma was transferred to thoracotomy due to extensive pleural adhesion during the operation. All patients were extubated in the recovery room. The average hospitalization days were 12 days, and there were no serious postoperative complications. The median follow-up time was 8 months. All patients were alive, and there was no record of 90 day re-admission. Among them, there was no recurrence of tumor patients.

## Discussion

As far as we are concerned, this is the first time to report the application experience of da Vinci's robotic surgery system in 26 patients with thoracic diseases in plateau areas. Compared with traditional open and thoracoscopic surgery, robot-assisted surgery has the advantages of high-definition three-dimensional vision, flexible manipulator, tremor filtering and so on (6). It can ensure the safety and oncology radical effect in the treatment of various diseases in thoracic surgery, and at the same time, it has the advantages of reducing bleeding, shortening hospitalization time and reducing complications (7). However, the application of robotic surgery in Tibet also faces some challenges. For example, the plateau environment puts forward certain requirements for the stable operation and maintenance of robot equipment; At the same time, the promotion and application of robot surgery technology also need to consider the training of local medical personnel and the improvement of technical level.

The average altitude in Tibet is over 4,000 m, and it is known as the “roof of the world”. The temperature in Tibet is small in annual range and the daily range is large due to the high terrain, thin air and strong solar radiation. The patients' pulmonary function is often poor and they are prone to bleeding during operation (8), which makes thoracic surgery more difficult than that in plain areas. Therefore, the rate of conversion in this article is slightly higher than that reported in previous papers (9). Because of calcification of lymph nodes around pulmonary artery, all of the surgeries were successfully completed by thoracoscope or thoracotomy without fatal complications, while robotic mediastinal and esophageal surgery was successfully completed. We believe that with the accumulation of experience, the rate of conversion will be lower and lower.

The original intention of robot-assisted surgery was to perform remote surgery on soldiers on the battlefield (10). Due to the high speed, high reliability, and low latency advantages of 5G networks, the clinical application of robotic surgery has accelerated significantly (11). Now China's 5G network technology is maturing, which could offer a new tool in improving care of patients requiring robotic surgical management such as tumor patients (12). Patients will not have to travel long distances, and they will be able to receive consultation surgery from domestic and foreign experts in local hospitals, and even a number of experts will cooperate to complete the surgery from a distance. For doctors, they can operate on patients in remote areas without leaving their work units, and the uneven distribution of medical resources will be greatly improved, which is particularly important for plateau areas like Tibet.

In conclusion, we believe that the application of da Vinci robotic surgery system in Tibet plateau should be actively carried out on the premise of careful and serious discussion of surgical indications.

## Data Availability

The original contributions presented in the study are included in the article/supplementary material, further inquiries can be directed to the corresponding author.
